# ATP as Phosphorus and Nitrogen Source for Nutrient Uptake by *Fagus sylvatica* and *Populus* x *canescens* Roots

**DOI:** 10.3389/fpls.2019.00378

**Published:** 2019-04-04

**Authors:** Ursula Scheerer, Niclas Trube, Florian Netzer, Heinz Rennenberg, Cornelia Herschbach

**Affiliations:** ^1^Chair of Tree Physiology, Institute of Forest Sciences, Albert-Ludwigs-University Freiburg, Freiburg, Germany; ^2^Chair of Ecosystem Physiology, Institute of Forest Sciences, Albert-Ludwigs-University Freiburg, Freiburg, Germany

**Keywords:** adenosine uptake, ADP/AMP uptake, ATP uptake, excised non-mycorrhizal roots, *Fagus sylvatica*, phosphatase inhibition, *Populus* x *canescens*, uptake competition

## Abstract

The present study elucidated whether roots of temperate forest trees can take up organic phosphorus in the form of ATP. Detached non-mycorrhizal roots of beech (*Fagus sylvatica*) and gray poplar (*Populus* x *canescens*) were exposed under controlled conditions to ^33^P-ATP and/or ^13^C/^15^N labeled ATP in the presence and absence of the acid phosphatase inhibitor MoO_4_^2-^. Accumulation of the respective label in the roots was used to calculate ^33^P, ^13^C and ^15^N uptake rates in ATP equivalents for comparison reason. The present data shown that a significant part of ATP was cleaved outside the roots before phosphate (P_i_) was taken up. Furthermore, nucleotide uptake seems more reasonable after cleavage of at least one P_i_ unit as ADP, AMP and/or as the nucleoside adenosine. Similar results were obtained when still attached mycorrhizal roots of adult beech trees and their natural regeneration of two forest stands were exposed to ATP in the presence or absence of MoO_4_^2-^. Cleavage of P_i_ from ATP by enzymes commonly present in the rhizosphere, such as extracellular acid phosphatases, ecto-apyrase and/or nucleotidases, prior ADP/AMP/adenosine uptake is highly probable but depended on the soil type and the pH of the soil solution. Although uptake of ATP/ADP/AMP cannot be excluded, uptake of the nucleoside adenosine without breakdown into its constituents ribose and adenine is highly evident. Based on the ^33^P, ^13^C, and ^15^N uptake rates calculated as equivalents of ATP the ‘pro and contra’ for the uptake of nucleotides and nucleosides is discussed.

Short Summary

Roots take up phosphorus from ATP as P_i_ after cleavage but might also take up ADP and/or AMP by yet unknown nucleotide transporter(s) because at least the nucleoside adenosine as N source is taken up without cleavage into its constituents ribose and adenine.

## Introduction

Phosphorus (P) is one of the six macronutrients in all living organism essential for growth and development due to its function in DNA and RNA for inheritance, in free nucleotides for energy transfer, in phospholipids as membrane components as well as in sugar phosphates within carbon metabolism including signaling and regulation processes. Different to nitrogen (N) and sulfur (S), which are acquired by plant roots from the soil *via* active uptake mechanisms (e.g., Gigolashvili and Kopriva, 2014; Rennenberg and Dannenmann, 2015; Castro-Rodríguez et al., 2017) and from the atmosphere *via* diffusion through the stomata of leaves (e.g., Gessler et al., 2000, 2002; Herschbach, 2003), P is exclusively available in the soil. With soil development (pedogenesis), the already low availability of P (Bieleski, 1973) further decreases due to long-term weathering, erosion, and leaching (Turner and Condron, 2013). P input into the soil by P deposition is extremely low (Peñuelas et al., 2013) and a chemical shift of plant available to unavailable organic bound phosphate (P_org_) (Walker and Syers, 1976; Callaway and Nadkarni, 1991; Chadwick et al., 1999; Vitousek et al., 2010; Vincent et al., 2013) further diminishes the plant available P in the soil. As a consequence, during plant evolution several morphological, physiological, and molecular strategies have been developed to overcome this limitation (Vance et al., 2003; Lambers et al., 2008, 2015a,b). P acquisition can be improved by the formation of cluster roots in *Proteaceae* at P limitation (Lambers et al., 2015a). Mycorrhizal association, evolved by about 90% of all land plants, largely enhances the root surface as well as the accessibility to small diameter soil pores; thereby mycorrhizal hyphae are the most important sites of P acquisition of most plant species (Jansa et al., 2011; Smith et al., 2015). Increased organic acid and acid phosphatase exudation improves P_i_ solubilization of Al- and Fe-bound P and the cleavage of organic-bound P, respectively (Tran et al., 2010; Chen and Liao, 2016).

A major part of soil phosphate (P_i_) is adsorbed to Fe and Al oxyhydroxides and, hence, is not available for plant uptake (Prietzel et al., 2016), but is also present as phosphate (di)esters such as nucleic acids, sugar phosphate and phospholipids as P_org_ (Plassard and Dell, 2010). Exudation of organic acids by the roots (Plaxton and Tran, 2011; Tian and Liao, 2015) supports phosphate (P_i_) solubilization from chelated aluminum- and iron-P (Hinsinger, 2001; Jones and Oburger, 2011; Marschner et al., 2011; Prietzel et al., 2016). Extracellular phosphatases produced and exuded by microbes, fungi and plant roots mediate P_i_ cleavage from P_org_ and make P_i_ from P_org_ available for the uptake by roots (Hinsinger et al., 2015; Smith et al., 2015; Tian and Liao, 2015). The release of acid phosphatase into the rhizosphere by microbes and plants depends on the soil-P level, with higher activity at P-poor than at P-rich forest soils (Hofmann et al., 2016). Furthermore, gross and net P mineralization was found to be negligible in soils developed on a P-rich basalt site, but biological and biochemical processes dominate P mineralization in a P-poor sandy soil (Bünemann et al., 2016). P_i_ uptake by plant roots is furthermore adapted to plant available P_i_ concentrations in the soil solution at the level of P_i_ transporter expression (Kavka and Polle, 2016). All these strategies and processes can influence and affect the acquisition of P_i_, the only form of P described to be taken up by plant roots (Chiou and Lin, 2011).

The amount of P_org_ in soils depends on soil type and age (Jones and Oburger, 2011). For example, about 95% of mobile P in a rendzic forest soil was found to be P_org_ (Kaiser et al., 2003). In this context, it is remarkable that mobilization of glucose-6-phosphate from ferrihydrite by ligand-promoted dissolution via organic acids, such as oxalate and ascorbate, is higher than mobilization of P_i_ (Goebel et al., 2017). Hence, P_org_ may be highly available in the rhizosphere after organic acid exudation. Furthermore, P acquisition by plants is mainly achieved from the organic layer by ectomycorrhizal fungi (Zavišić et al., 2016). In the organic soil layer plant available P_i_ was 5 to 36 times higher than in the mineral layer. However, in the organic soil layer most of the total P was found to be attributed to P_org_ fixed in plant litter and living organism of the rhizosphere and only 10–24% was present as P_i_ (Zavišić et al., 2016; Lang et al., 2017). Hence, P_org_ is an important P source that gets available during degradation of root and leaf litter as well as dead microbes and soil organic matter (SOM) (Shen et al., 2011).

Altogether, this summary indicates the importance of P acquisition from P_org_ by plant roots. However, the preferential P_org_ compound(s) used in P_i_ release (e.g., nucleotides *versus* sugar phosphates) by acid and alkaline phosphatases, the significance of P_i_ release *versus* direct P_org_ uptake, and the interaction/competition between P_i_ and P_org_ for P_i_ uptake by the roots have not been established. Such interactions were found for the inorganic and organic N uptake by the roots of woody plants (e.g., Stoelken et al., 2010). Determination of ATP in the soil is frequently used to quantify microbial biomass (Blagodatskaya and Kuzyakov, 2013) and, consequently, ATP seems to be available for P acquisition by the roots. In addition, extracellular ATP mostly correlated with regions of active growth and cell expansion and has been discussed as a signal in growth control (Kim et al., 2006; Tanaka et al., 2010, 2014; Yang et al., 2015). Hence, mobility of ATP across the root plasma membrane is highly probable. Consequently, roots might take up ATP and other P_org_ compounds such as sugar-Ps. Although the significance of P_org_ as P_i_ source for P nutrition of plants is well known (e.g., Thomas et al., 1999; Liang et al., 2010), direct uptake of P_org_ compounds has not been established.

The aim of the present study was to elucidate, if roots of temperate forest trees can take up P_org_ in the form of ATP. We hypothesized that ATP and/or one of its degradation products ADP, AMP, as important P_org_ compounds of soil, root and leaf litter, and of microbial detritus in the rhizosphere, can be taken up by tree roots as intact molecule. We further hypothesized that ATP and P_i_ uptake compete with each other. These hypotheses were tested under controlled conditions with detached roots of two temperate forest tree species colonizing different ecological niches; i.e., beech (*Fagus sylvatica*) the most important climax tree species of Central European temperate forests and poplar (*Populus* x *canescens*) a continuously, fast growing tree species of floodplains (Stimm and Weisgerber, 2008).

## Materials and Methods

### Plant Material for Experiments Under Controlled Conditions

Poplar cuttings (*Populus tremula* × *Populus alba*, synonym *Populus* x *canescens*) of the INRA clone 717 1B4) were micropropagated (Strohm et al., 1995), transferred into sand after 4–6 weeks of growth (Herschbach et al., 2010; Scheerer et al., 2010; Honsel et al., 2012) and cultivated in a greenhouse under long-day conditions for further 14–18 weeks. Poplar plants were fertilized with 200 mL modified ¼ Hoagland solution per week (Herschbach et al., 2010; Honsel et al., 2012). The one fourth modified Hoagland solution (Hoagland and Arnon, 1950) contained: 0.6 mM KNO_3_, 1.3 mM Ca(NO_3_)_2_ × 4 H_2_O, 0.3 mM MgSO_4_ × 7 H_2_O, 1.5 mM MgCl_2_ × 6 H_2_O, 0.25 mM KH_2_PO_4_, 2.3 μM MnCl_2_ × 4 H_2_O, 2 μM H_3_BO_3_, 0.08 μM CuCl_2_ × 4 H_2_O, 0.2 μM ZnCl_2_, 0.2 μM Na_2_MoO_4_ × 2 H_2_O, 0.04 μM CoCl_2_ × 6 H_2_O, 22.5 μM Na_2_-EDTA, 22.5 μM FeCl_2_ and was adjusted to pH 5.5. If necessary poplar plants were provided with distilled water.

Beech seedlings were cultivated from beech nuts collected in 2011 from the Conventwald forest stand (Forstbaumschule Stingl, Albstadt-Burgfelden, Germany) [7.960 East; 48°02′ North (Google earth); von Wilpert et al., 1996; Netzer et al., 2017] and stored for stratification at 8°C. Beech nuts were germinated as described in detail by Kreuzwieser et al. (1996). Briefly: nuts were soaked in tap water for 4 weeks at 4°C. After germination, seeds were peeled, surface sterilized and kept for 2–4 weeks at axenic conditions. Thereafter, seedlings were transferred into a sand (particle size 1–2 mm)/vermiculite (1:1) mixture in pots of 1 L size. Beech seedlings were fertilized two times a week with 100 mL of a nutrient solution adapted to the soil water of the Conventwald forest (Netzer et al., 2017). This solution contained 290 μM NH_4_Cl, 350 μM KNO_3_, 160 μM CaCl_2_, 170 μM MgSO_4_, 20 μM KH_2_PO_4_, 0.23 μM MnCl_2_, 0.02 μM ZnCl_2_, 0.2 μM H_3_BO_3_, 0.008 μM CuCl_2_, 0.02 μM Na_2_MoO_4_, 0.004 μM CoCl_2_, and 2.25 μM FeCl_2_ and was adjusted to pH 5.5. Beech seedlings were grown for more than 3 months in a greenhouse under long-day conditions. In addition, 2-year old beech seedlings from a commercial supplier (Eberts OHG, Tangstedt/Pbg., Germany) were used.

### Measurements of ^33^P Uptake Applied as ^33^P-PO_4_^3-^ and ^33^P-ATP

For uptake measurement of ^33^P-P_i_ (Hartmann Analytic, Braunschweig, Germany), roots were excised from *P.* x *canescens* plants, which were 14 to 18-weeks old and/or 0.7–1 m in height (Herschbach et al., 2010; Honsel et al., 2012). Roots of beech seedlings were excised after removing vermiculite and peat particles. Excised roots of both species were placed into an incubation chamber (Herschbach and Rennenberg, 1991), which consisted of three compartments, i.e., an application compartment (compartment A, 50 mL), a buffer compartment (compartment B, 20 mL) and a compartment for xylem sap exudation (compartment C, 30 mL). In case of poplar, for pre-incubation the compartments were filled with ¼ Hoagland solution (compartment B and C without P_i_) supplemented with 2 mM MES buffer and adjusted to pH 5.0. In case of ^33^P-ATP treatments, the respective (pre-) incubation solutions in compartment A did not contain phosphate and molybdate but ATP. Beech roots were pre-incubated in the beech fertilization solution supplemented with 2 mM MES buffer adjusted to pH 5.0. The pH dependency of P_i_ uptake was analyzed with excised poplar roots over a range of pH 3.5 to pH 7 (Hinsinger, 2001) and revealed highest values at pH 4.5 to pH 5.5, but no marked pH optimum (Supplementary Figure S1). Hence, all uptake experiments were performed at pH 5.0.

**FIGURE 1:**
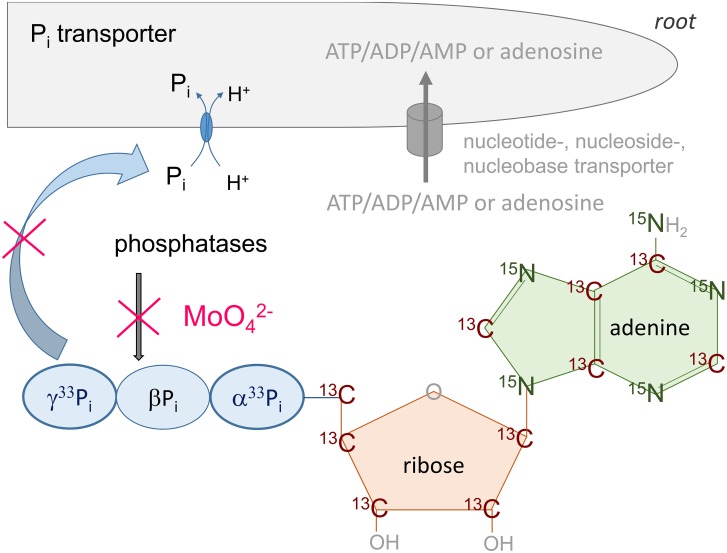
Overview of the experimental designs with differently labeled ATP molecules. During the experiments, three differently labeled ATP molecules were applied: α^33^P-ATP; γ^33^P-ATP, ^13^C/^15^N labeled ATP (ATP ^13^C_10_H_16_^15^N_5_O_13_P_3_ xNa) with the ^13^C label in the ribose and base. The base adenine/cytidine was additionally labeled by ^15^N. Molybdate was applied as a common acid phosphatase inhibitor to prevent cleavage of the γP_i_ and βP_i_ unit of the ATP molecule (Gallagher and Leonard, 1982; Cabello-Díaz et al., 2012). Uptake of nucleotides such as ATP, ADP, AMP and/or adenosine via yet unknown transporters is indicated. Unlabelled phosphate (P_i_) in the solution competed with the P_i_ cleaved from ATP by extracellular phosphatases, ecto-apyrases, or nucleotidases (Wu et al., 2007; Riewe et al., 2008; Tanaka et al., 2014) for the uptake via P_i_ uptake transporters.

Incubation chambers were placed on aluminum plates cooled down to 15°C to simulate soil temperature. Excised roots of beech and poplar were pre-incubated for 2 h (Herschbach et al., 2010). After pre-incubation the solution of the application compartment (compartment A) was replaced by the respective solution supplemented with radiolabeled 0.25 mM ^33^P-phosphate (4.1^∗^10^7^ to 5.3^∗^10^7^ Bq mmol^-1^ P_i_) or with 0.169 mM ^33^P-ATP (5.3^∗^10^7^ to 1.2^∗^10^7^ Bq mmol^-1^ ATP). ^33^P-ATP was applied either as γ^33^P-ATP or as α^33^P-ATP (Figure 1). Uptake of ^33^P from ^33^P-P_i_ and ^33^P-ATP was terminated after 4 h [during this time, linear uptake can be assumed (Herschbach and Rennenberg, 1991)] by washing the roots three-times with the respective unlabeled solution to remove adherent labeled compounds. Root sections of the incubation compartment were separated from the root part located in compartment B and C. ^33^P was determined by liquid scintillation counting after sample bleaching as previously described (Herschbach et al., 2010; Scheerer et al., 2010). Calculation of uptake rates as well as of xylem loading rates was performed according to Herschbach and Rennenberg (1991).

### Experiments of ^13^C and ^15^N Uptake Applied as Double-Labeled ATP and CTP Under Controlled Conditions

For uptake experiments with stable isotope labeled ATP/CTP (Figure 1), excised roots of poplar and beech were placed into an incubation chamber (Herschbach and Rennenberg, 1991) consisting of an application compartment (compartment A; 85 mL), a buffer compartment (compartment B) and a xylem sap exudation compartment (compartment C) (each 10 mL). Double-labeled ATP (ATP-^13^C_10_^15^N_5_, 98 atom%, Sigma Aldrich) (Figure 1) and CTP (CTP-^13^C_9_^15^N_3_, 98 atom%, Sigma Aldrich) were diluted to 10 atom% or 14 atom% and were adjusted to the final concentration of 0.169 mM ATP and CTP. The soil microbial ATP concentration of active and dead microorganism, which are constituents of the rhizosphere, ranged from <1.2 μg g^-1^ soil (<5–10 μmol g^-1^ dormant microbial biomass) to >2 μg g^-1^ soil (>12–15 μmol g^-1^ active microbial biomass) (Blagodatskaya and Kuzyakov, 2013). The ATP concentration applied in the incubation solution corresponds to approximately 90 μg ATP mL^-1^ or to 0.169 mmol mL^-1^, which was in the range of several experiments performed to test physiological responses to extracellular ATP (Roux and Steinebrunner, 2007). Roots were pre-incubated with the respective solutions without phosphate and molybdate. After 2 h of pre-incubation the incubation solution of compartment A was replaced by the respective solution that contained 0.169 mM of ^13^C/^15^N labeled ATP or CTP (10 or 14 atom%). To simulate soil temperature, incubation chambers were placed on aluminum plates cooled down to 15°C for 4 h of incubation. Uptake of ATP or CTP was terminated by washing roots 3-times with the respective unlabeled solution. Root sections in the incubation compartment (compartment A) were separated from the root sections located in compartment B and C. Oven dried homogenized root samples were subjected to IRMS analysis for the determination of ^13^C and ^15^N accumulation.

### Experiments of ^13^C and ^15^N Uptake Applied as Double-Labeled ATP in the Field

To test if ^13^C and ^15^N uptake rates calculated as ATP equivalents in experiments under controlled conditions were similar to ^13^C and ^15^N uptake rates equivalent to ATP in the field, ATP uptake experiments were performed in September 2017 at two field sites, namely the acidic Conventwald (Con) and calcareous Tuttlingen (Tut) forest stands. The soils of these forests differ in their properties (silicate *versus* limestone bedrock) (Prietzel et al., 2016) with the Tuttlingen soil containing eightfold lower plant available P_i_ (for detailed soil descriptions see Prietzel et al., 2016; Netzer et al., 2017). At both field sites, fine roots of six adult beech trees and of six beech saplings were carefully excavated out of the soil. Adherent soil particles from the roots were removed with distilled water and cleaned roots were dried using paper towels. Roots still attached to adult beech trees or to their offspring were incubated in an artificial soil solution at pH 5.0 that contained 29 μM NH_4_Cl, 35 μM KNO_3_, 16 μM CaCl_2_, 17 μM MgCl_2_ 0.3 μM MnCl_2_, 22 μM NaCl, and 0.169 mM ATP. Double-labeled ATP was diluted to 10 atom% (ATP-^13^C_10_^15^N_5_, 98 atom%, Sigma Aldrich). Fine roots were cut from the trees after 4 h of incubation, rinsed with distilled water to remove adherent ATP-^13^C_10_^15^N_5_, dried in an oven (72 h, 50°C) for at least 2 days and homogenized using mortar and pestle.

### Analysis of C and N Contents and of the ^13^C and ^15^N Abundance

^13^C and ^15^N incorporation into root sections after ATP-^13^C_10_^15^N_5_ and CTP-^13^C_9_^15^N_3_ exposure were determined in over dried powdered root samples of 0.1–2.0 mg aliquots filled into tin capsules (Hu et al., 2017). Total carbon and nitrogen contents as well as the ^15^N and ^13^C abundances were determined using an elemental analyzer (NA 2500CE Instruments, Milan, Italy) coupled via a Conflo II interface to an isotope ratio mass spectrometer (Delta Plus, Thermo Finnigan MAT GmbH, Bremen, Germany). Alternatively, samples were analyzed with an elemental analyzer NA 1108, Fisons-Instruments, Rodano, Milan, Italy and a mass spectrometer (Delta C, Finnigan MAT, Bremen, Germany) coupled by a ConFlo III interface (Thermo Electron Corporation, Bremen, Germany) (Zieger et al., 2017). A working standard (glutamic acid) was calibrated against the primary standards of the United States Geological Survey 40 (USGS 40; glutamic acid δ^13^ CPDB = -26.39‰) and USGS 41 (glutamic acid δ^13^ CPDB = 37.63‰) for δ^13^C, and USGS 40 (glutamic acid δ^15^ N_air_ = -4.5‰) and USGS 41 (glutamic acid δ^15^ N_air_ = 47.600‰) for δ^15^ N. Standards were analyzed after every tenth sample to account for potential instrument drift over time as described by Dannenmann et al. (2009) and Simon et al. (2011). Accumulation of δ^15^N and δ^13^C was used to calculate N and C uptake rates in equivalents of ATP and CTP (Rennenberg et al., 1996; Gessler et al., 1998).

### Data Analyses

For comparison, uptake of ^33^P as well as of ^13^C and ^15^N from differently labeled ATP/CTP was calculated from ^33^P, ^15^N and ^13^C incorporation as equivalents of ATP. This standardized calculation allows direct comparison between treatments and uncovers differences between the differently labeled ATP (Figure 1). Statistical analyses were performed with Origin^®^9.1 (OriginLab Corporation^1^). Normal distribution of the data was tested with the Shapiro–Wilk and Kolmogorov–Smirnov test; all data showed normal distribution at least by the Kolmogorov–Smirnov test. One Way ANOVA was applied followed by the Bonferroni and Tukey test with *p* < 0.5. Data are presented as single values (left to the box plots) and as box-plots showing the median (black line), the mean (open square), and the 25 and 75 percentile. Minimum and maximum values are given as error bars, whereas outliers (1%) are presented as stars.

## Results

### Competition of P_i_ Uptake by ATP

**FIGURE 2:**
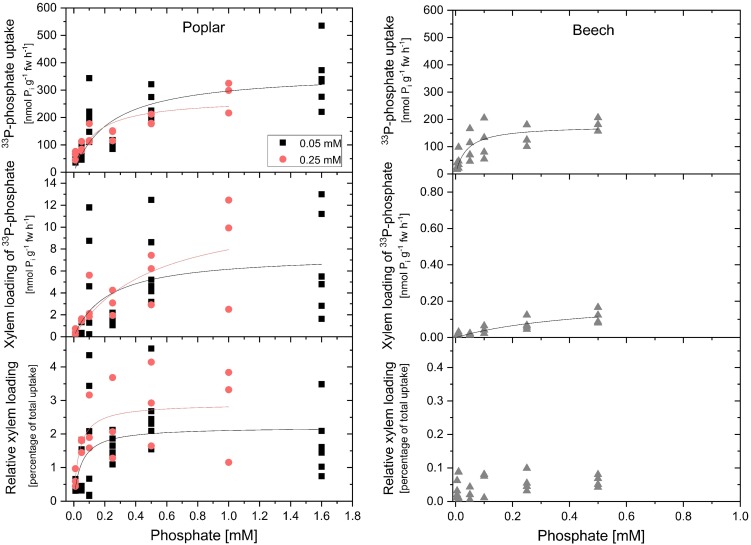
Concentration dependency of phosphate uptake, xylem loading of phosphate and the relative proportion of phosphate loaded into the xylem of excised poplar and beech roots. Concentration dependency of phosphate (P_i_) uptake (upper graphs), xylem loading of phosphate (middle graphs) and the relative proportion of phosphate loaded into the xylem (bottom graphs) was performed with excised poplar (left column) and beech (right column) roots. Poplar plants were grown either with 0.05 mM P_i_ (black squares) or with 0.25 mM P_i_ (red dots). Beech seedlings were cultivated with 0.02 mM P_i_. Data presented are values from individual incubations with four to six excised roots. Michaelis–Menten fits were calculated using the data analysis and graphic software Origin^®^9.1. The black and red curves show Michaelis–Menten fits for the respective plant sets; black: growth *P*_i_ = 0.05 mM; red: growth *P*_i_ = 0.25 mM for poplar, and gray for beech. After 2 h of pre-incubation the 4 h of incubation were started by replacing the solution of the incubation compartment with the respective incubation solution containing the P_i_ concentration indicated; for poplar from 0.01 mM up to 1.6 mM P_i_ and for beech from 5 μM up to 0.5 mM P_i_. Specific activity of ^33^P-P_i_ ranged from ∼2.0^∗^10^8^ Bq mmol^-1^ (application of 0.05 mM P_i_) up to ∼5.7^∗^10^6^ Bq mmol^-1^ (treatment of 1.6 mM P_i_) for poplar and ranged from ∼1.9^∗^10^9^ Bq mmol^-1^ (application of 0.005 mM P_i_) up to ∼1.9^∗^10^7^ Bq mmol^-1^ (treatment of 0.5 mM P_i_) for excised beech roots.

P_i_ uptake rates of excised roots calculated from ^33^P-P_i_ application (compare Figure 1) for both, poplar and beech, followed Michaelis–Menten kinetics (Figure 2). Growth P_i_ concentration only slightly affected *K*_m_ and *v*_max_ values of P_i_ uptake of excised poplar roots. At 0.25 mM growth P_i_, a marginally higher P_i_ affinity was indicated by a lower *K*_m_-value (126 ± 49 μM) compared to growth at 0.05 mM P_i_ (*K*_m_ value of 238 ± 94 μM). The maximum P_i_ uptake rate was lower during growth at 0.25 mM P_i_ (271 ± 31 nmol g^-1^ fw h^-1^) compared to the growth at 0.05 mM P_i_ (367 ± 50 nmol g^-1^ fw h^-1^). Excised roots from beech seedlings cultivated with 0.02 mM P_i_ showed remarkably lower *K*_m_ (39 ± 18 μM) and *v*_max_ values (178 ± 21 nmol g^-1^ fw h^-1^). The tripartite incubation chamber allowed calculation of the P_i_ that has been loaded into the xylem (Herschbach and Rennenberg, 1991). Growth P_i_ did not affect this parameter that accounts for up to 4% of total P_i_ taken up by excised roots for poplar (Figure 2). In contrast, the P_i_ loaded into the xylem of excised beech roots was extremely low and reached approximately 0.1% of total P_i_ taken up that was close to the limit of detection (Figure 2). Maximum rate of P_i_ loaded into the xylem was 13 nmol g^-1^ fw h^-1^ for poplar but only 0.17 nmol g^-1^ fw h^-1^ for beech.

**FIGURE 3:**
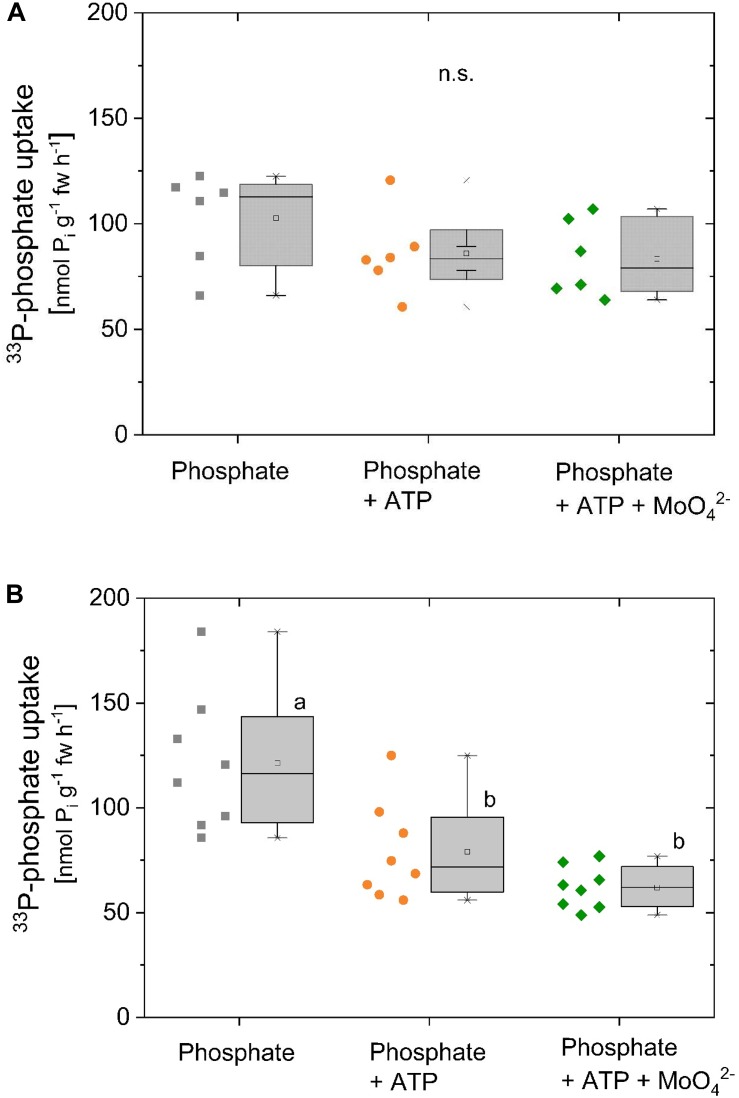
Competition of P_i_ uptake by ATP of excised poplar and beech roots. Competition of P_i_ uptake by ATP was investigated with excised poplar (**A**, *n* = 6) and excised beech (**B**, *n* = 8) roots. **(A)** Roots were excised from poplar plants grown with 0.25 mM P_i_ (experiments were performed in October). During the incubation 0.5 mM ^33^P-P_i_ (∼2.8^∗^10^7^ Bq mmol^-1^) was applied either solely, together with 0.338 mM ATP or together with ATP plus the acid phosphatase inhibitor MoO_4_^2-^ (0.5 mM). **(B)** Roots were excised from beech seedlings cultivated with 0.02 mM P_i_ (experiments were performed in December/January). During incubation 0.25 mM ^33^P-P_i_ (∼4.5^∗^10^7^ Bq mmol^-1^) was applied either solely, together with 0.25 mM ATP or together with ATP plus the acid phosphatase inhibitor MoO_4_^2-^ (0.5 mM). Data are presented as box plots with individual data left to the box plots. Different small letters indicate significant differences between treatments at *p* < 0.05 analyzed by One Way ANOVA followed by the *Post hoc* tests Bonferroni and Tukey.

^33^P-P_i_ uptake by excised poplar roots remained unaffected by the presence of ATP (Figure 3A). Application of MoO_4_^2-^, a common inhibitor of acid phosphatases, except for intracellular phosphatases, was used to prevent P_i_ cleavage from ATP (Gallagher and Leonard, 1982; Bozzo et al., 2002; Cabello-Díaz et al., 2012). By applying molybdate, dilution of the specific activity of ^33^P-P_i_ by unlabelled P_i_ cleaved from ATP was supposed to be prevented. Under these conditions, ^33^P-P_i_ uptake of excised poplar roots was also not affected if ATP was present (Figure 3A). Xylem loading of phosphate in this experiment was below 2 nmol g^-1^ fw h^-1^ (data not shown). In contrast, ^33^P-P_i_ uptake of excised beech roots significantly declined in the presence of ATP (Figure 3B). However, addition of MoO_4_^2-^ to prevent P_i_ cleavage from ATP did not recover ^33^P-P_i_ uptake by excised beech roots. Apparently, the decline in ^33^P-P_i_ uptake by excised beech roots in the presence of ATP was not a dilution effect by ATP cleavage through acid phosphatases but could be due to the cleavage through ecto-apyrases. Xylem loading of ^33^P-P_i_ was below 0.2 nmol g^-1^ fw h^-1^ (data not shown).

### ^33^P Uptake From γ^33^P-ATP

**FIGURE 4:**
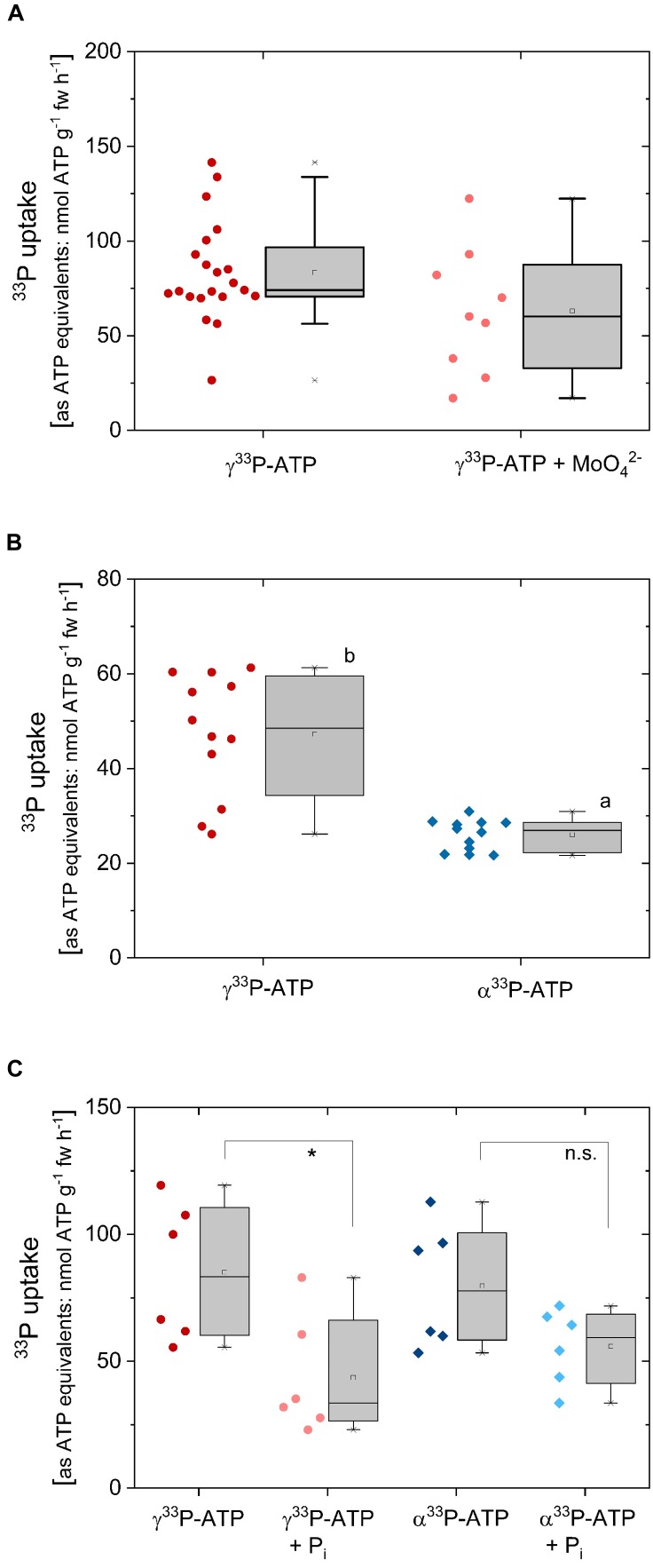
^33^P uptake rates as ATP equivalents of excised poplar roots, its competition by P_i_ and the effect of acid phosphatase inhibition. Roots were excised from poplar plants cultivated with 0.25 mM P_i_. **(A)** Excised poplar roots were incubated with γ^33^P-ATP (0.169 mM; ∼6.0^∗^10^7^ Bq mmol^-1^) either solely of together with the acid phosphatase inhibitor MoO_4_^2-^ (0.5 mM). The experiments (*n* = 21 biological replicates treated without molybdate and 9 biological replicates treated with molybdate) were performed during February/March. **(B)** Excised poplar roots were incubated with γ^33^P-ATP or α^33^P-ATP (0.169 mM; 4.2^∗^10^7^ to 6.4^∗^10^7^ Bq mmol^-1^) either solely (**B**, *n* = 12; experiments were carried out end of March/at the beginning of April) or in combination with 0.25 mM P_i_ for competition (**C**, *n* = 6, experiments were carried out in May). In order to be able to compare ^13^C and ^15^N uptake rates, both were calculated as ATP equivalents, i.e., 5 ^15^N correspond to one ATP while 10 ^13^C are equivalent to one ATP. Data are presented as box plots with individual data left to the box plots. Different small letters indicate significant differences at *p* < 0.05 between treatments analyzed by One Way ANOVA followed by the *Post hoc* tests Bonferroni and Tukey. The asterisk in C indicates significant differences between the treatments γ^33^P-ATP and γ^33^P-ATP plus P_i_ (*p* < 0.05). Variation of ^33^P uptake rates as ATP equivalents between different experiments presented here and in Figure 6A may be due to seasonal variations as observed for P_i_ (Netzer et al., 2018).

Uptake of ^33^P from γ^33^P-ATP by excised poplar roots was determined as ^33^P incorporation and calculated as ATP equivalents (approximately 83 ± 27 nmol ATP g^-1^ fw h^-1^) (Figure 4A). ^33^P from γ^33^P-ATP can be taken up as ATP, but also as ^33^P-P_i_ after cleavage by phosphatases. Application of the acid phosphatase inhibitor MoO_4_^2-^ slightly, but not significantly, diminished ^33^P incorporation that amounted 63 ± 33 nmol g^-1^ fw h^-1^ in the presence of MoO_4_^2-^ (Figure 4A). Xylem loading of ^33^P from the applied γ^33^P-ATP was significantly lower in the presence of MoO_4_^2-^ and amounted 0.3 ± 0.2 nmol g^-1^ fw h^-1^ compared to 0.6 ± 0.3 nmol g^-1^ fw h^-1^ in the absence of MoO_4_^2-^.

Another approach to test the importance of P_i_ cleavage for the uptake of ^33^P from ^33^P labeled ATP was tested by comparing ^33^P uptake from γ^33^P-ATP and α^33^P-ATP. Assuming that ^33^P prior its uptake must be cleaved from ATP by phosphatases, ^33^P uptake should be lower when the α-P instead of the end standing γ-P was labeled as ^33^P. Indeed, ^33^P uptake was significantly lower when the α-P instead of the γ^33^P in the ATP was labeled (Figure 4B). Hence, it can be assumed that poplar roots take up part of the ^33^P as P_i_ after cleavage from γ^33^P-ATP by phosphatases and/or ecto-apyrases. To test this assumption, P_i_ was added to the incubation solutions together with γ^33^P-ATP and α^33^P-ATP. It was expected that the non-labeled P_i_ diluted the ^33^P_i_ signal in excised poplar roots to a higher extent when ATP was applied as γ^33^P-ATP compared to the application of α^33^P-ATP. As expected, P_i_ significantly diminished the ^33^P incorporation into excised roots from γ^33^P-ATP, but not from α^33^P-ATP (Figure 4C). The xylem loading rate of ^33^P-P_i_ was below 1 nmol g^-1^ fw h^-1^ and was not affected by P_i_ supplementation (data not shown).

### ^13^C and ^15^N Uptake From Labeled ATP by Excised Poplar Roots

**FIGURE 5:**
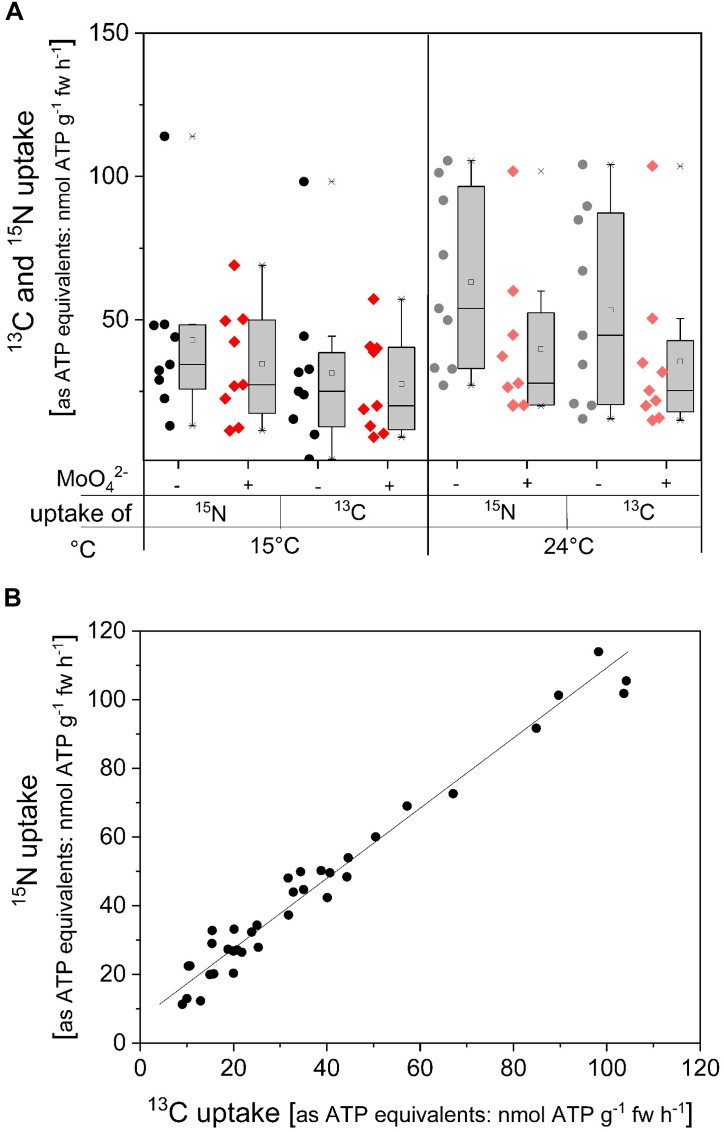
^13^C and ^15^N uptake rates in ATP equivalents of excised poplar roots in dependency on temperature and on acid phosphatase inhibition by MoO_4_^2-^. Roots were excised from poplar plants cultivated with 0.25 mM P_i_. **(A)** Excised poplar roots were incubated with 0.169 mM ATP (14 atom% ATP-^13^C_10_^15^N_5_) at 15°C (*n* = 9) or at 24°C (*n* = 9) either solely or together with the acid phosphatase inhibitor MoO_4_^2-^ (0.5 mM). Data from experiments performed in June/July are presented as box plots with individual data left to the box plots. In order to be able to compare ^13^C and ^15^N uptake rates, both were calculated as ATP equivalents, i.e., five ^15^N correspond to 1 ATP while 10 ^13^C are equivalent to one ATP. Significant differences were analyzed with the non-parametric Kruskal–Wallis test because normal distribution of the data was not given. Nevertheless, significant differences were not found at *p* < 0.05. **(B)** Correlation between ^13^C and ^15^N uptake rates as ATP equivalents of all individual incubation chambers, i.e., all samples and treatments. Correlation characteristics are given in Table 1.

^13^C/^15^N labeled ATP was applied as another approach to investigate ATP uptake. In the ^13^C/^15^N labeled ATP, ribose was labeled only with ^13^C whereas adenine was labeled by both, ^13^C and ^15^N. In order to compare ^13^C and ^15^N uptake rates both were calculated as ATP equivalents, i.e., five ^15^N correspond for one ATP, while ten ^13^C are equivalent to one ATP. Incubation with doubled labeled ATP at 15°C, applied to simulate soil temperature in forest stands, resulted in similar ^13^C and ^15^N uptake rates equivalent to ATP and were not affected by the acid phosphatase inhibitor MoO_4_^2-^ (Figure 5). Xylem loading of ^15^C and ^15^N in this approach was below the detection limit. At higher incubation temperature ^13^C and ^15^N uptake rates as ATP equivalents were slightly increased, however, this increase was not statistically significant. Inhibition of acid phosphatases by MoO_4_^2-^ slightly diminished ^13^C and ^15^N uptake rates calculated as ATP equivalents, but again this decline was not statistically significant. These results indicate that at least one P_i_ unit needs to be cleaved before roots can take up resulting ADP, AMP and/or adenosine. The strong correlation between ^13^C and ^15^N uptake rates as ATP equivalents suggest that ribose was taken up together with the adenine base (Table 1 and Figure 5B).

**Table 1:** Correlation analyses between ^13^C uptake (x-axis) and ^15^N or ^33^P uptake rates calculated as ATP/CTP equivalents.

*y*-axis		Slope	*y*-intercept	*P*	*r*^2^	Figure
As ATP/CTP equivalents	In the presence of:					
^15^N uptake	^13^C/^15^N-ATP plus α^33^P-ATP	1.05 ± 0.09	12 ± 7	0.969	0.938	6C
^15^N uptake	^13^C/^15^N-ATP plus γ^33^P-ATP	0.95 ± 0.05	19 ± 5	0.989	0.978	6C
^33^P uptake	^13^C/^15^N-ATP plus α^33^P-ATP	1.64 ± 0.37	78 ± 31	0.817	0.668	6C
^33^P uptake	^13^C/^15^N-ATP plus γ^33^P-ATP	1.7 ± 0.9	255 ± 95	0.514	0.264	6C
^15^N uptake	^13^C/^15^N-ATP	1.02 ± 0.03	11 ± 2	0.995	0.990	7C
^15^N uptake	^13^C/^15^N-CTP	0.89 ± 0.04	3.8 ± 2.7	0.990	0.980	7D
^15^N uptake	^13^C/^15^N-ATP	0.99 ± 0.03	8.3 ± 1.4	0.984	0.968	5B
^15^N uptake	^13^C/^15^N-ATP	0.91 ± 0.05	28 ± 7	0.936	0.876	8B


### Comparison of ^13^C, ^15^N and ^33^P Uptake Rates Applied as Triple Labeled ATP Experiment

**FIGURE 6:**
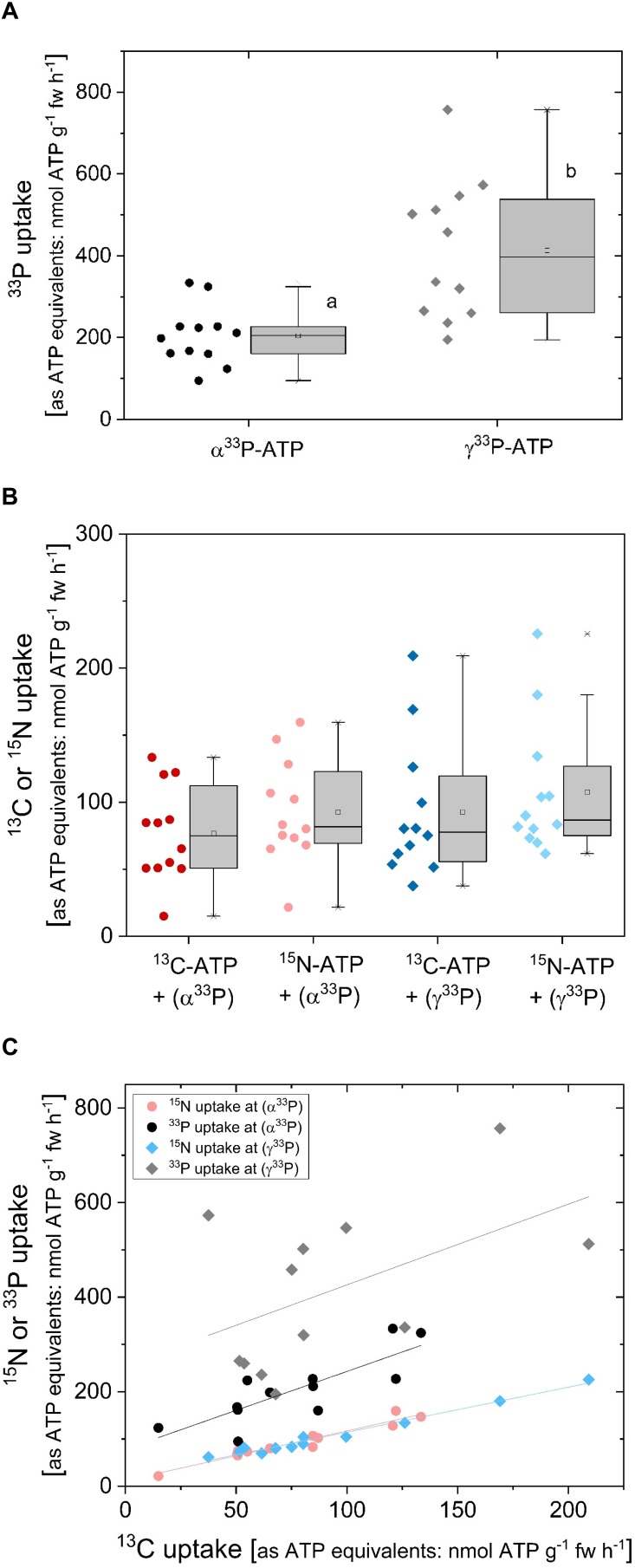
Comparison of ^33^P, ^13^C, and ^15^N uptake rates as ATP equivalents of excised poplar roots applied as ^33^P and ^13^C/^15^N labeled ATP. Uptake experiments (*n* = 12) were carried out in late autumn, i.e., at the beginning of November by applying α^33^P-ATP or γ^33^P-ATP (∼5.3^∗^10^7^ Bq mmol^-1^) together with ^13^C/^15^N labeled ATP (ATP-^13^C_10_^15^N_5_; 10 atom%) at the final concentration of 0.169 mM ATP. **(A)** ^33^P uptake rates were calculated in ATP equivalents. The rate of ^33^P loaded into the xylem was calculated from ^33^P incorporation and amounted to 2.5 ± 1.1 nmol g^-1^ fw h^-1^ calculated as ATP equivalents in the case of α^33^P-ATP and to 8.3 ± 2.3 nmol g^-1^fw h^-1^ in the case of γ^33^P-ATP application. This corresponds to 1.5 ± 1.4 and 2.2 ± 0.9% of the ^33^P that was loaded into the xylem for the α^33^P-ATP and γ^33^P-ATP application, respectively. **(B)** ^13^C and ^15^N uptake rates as ATP equivalents either for the γ^33^P-ATP or α^33^P-ATP treatment, respectively. **(C)** Correlation between ^33^P and ^15^N uptake rates as ATP equivalents and the respective ^13^C uptake rates as ATP equivalents. Correlation characteristics are given in Table 1. Significant differences were marked with different small letters (*p* < 0.05) and were analyzed by One Way ANOVA followed by the *Post hoc* tests Bonferroni and Tukey.

The correlation of ^33^P uptake rates with ^13^C and ^15^N uptake rates was investigated in a triple labeling approach (Figure 6) as a further approach to test for ATP uptake as an intact molecule. In this experiment, ^33^P-ATP was applied together with 10 atom% ATP-^13^C_10_^15^N_5_. As already observed in ^33^P-ATP labeling experiments (Figure 4B), ^33^P uptake rates equivalent to ATP were significantly lower when α^33^P-ATP instead of γ^33^P-ATP was applied (Figure 6A). The approximately 10-fold higher ^33^P uptake rates in this experiment (Figure 6A, carried out in November) compared to the experiment presented in Figure 4B (carried out in spring) may be due to seasonal differences, which have already been observed for P_i_ uptake under controlled conditions (Netzer et al., 2018). Both, ^13^C and ^15^N uptake rates equivalent to ATP were twofold lower compared to the ^33^P uptake rate equivalent to ATP when α^33^P-ATP and, approximately fourfold lower compared to the ^33^P uptake rate equivalent to ATP when γ^33^P-ATP was applied (Figure 6B). Correlation analyses were performed to elucidate the relationships between uptake rates equivalent to ATP calculated from ^13^C, ^15^N and ^33^P incorporation. ^13^C and ^15^N uptake rates as ATP equivalents showed a strong correlation of 1.05 ± 0.09 (Table 1 and Figure 6C). ^13^C uptake rates shown less but still significant correlation to ^33^P uptake rates of 1.64 ± 0.39 (*P* = 0.817, *r*^2^ = 0.668, *y* intercept = 78 ± 31) when α^33^P-ATP and of 1.71 ± 0.90 (*P* = 0.514, *r*^2^ = 0.264, *y* intercept = 255 ± 95) when γ^33^P-ATP was applied. These results support the view of an uptake of AMP and the nucleoside adenosine by the roots.

### ^13^C and ^15^N Uptake Rates Were Similar From ^13^C/^15^N-Labeled ATP and CTP

**FIGURE 7:**
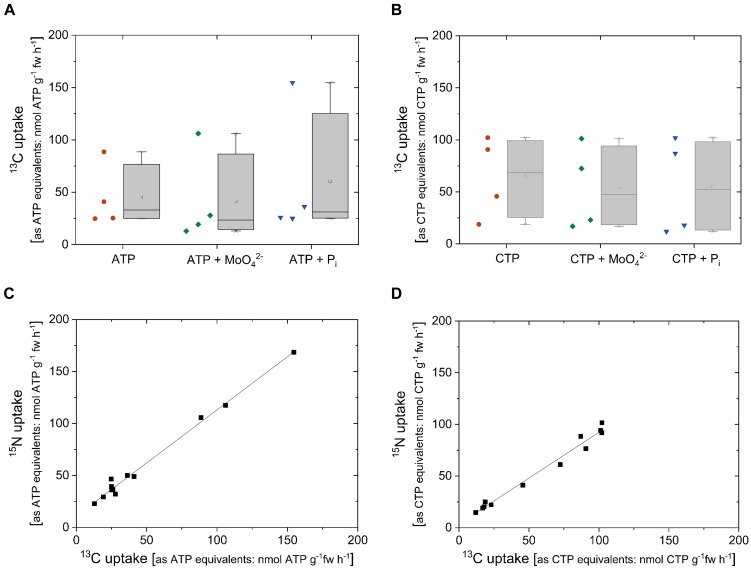
^13^C and^15^N uptake rates as ATP/CTP equivalents of excised poplar roots achieved from ^13^C/^15^N labeled ATP or CTP. Excised poplar roots were taken from poplar plants grown with 0.25 mM P_i_ (July). ^13^C and ^15^N uptake rates calculated as ATP and CTP equivalents were analyzed by the application of 0.169 mM labeled ATP-^13^C_10_^15^N_5_ (10 atom%) or CTP-^13^C_9_^15^N_3_ (10 atom%). The competition with P_i_ was tested by the addition of 0.25 mM P_i_. The effect of acid phosphatases was investigated by the simultaneous application of nucleotides plus 0.5 mM MoO_4_^2-^. Data presented are box plots for ^13^C uptake rates as ATP equivalents **(A)** and ^13^C uptake rates as CTP equivalents **(B)**. Left to the box plots individual values achieved from single incubation cambers are presented (*n* = 4). ^15^N uptake rates as ATP and CTP equivalents were similar to the ^13^C uptake rates as ATP and CTP equivalents. Significant differences were analyzed by One Way ANOVA followed by the *Post hoc* tests Bonferroni and Tukey with *p* < 0.05 but, statistically significant differences were not found. Correlations over all treatments between ^13^C and ^15^N uptake rates are provided for ATP **(C)** and CTP **(D)**. Slopes and Pearson correlation coefficients are given in Table 1.

To address the question whether excised poplar roots can take up ribose together with the base from other nucleotides, cytosine triphosphate (CTP) was applied as CTP-^13^C_9_^15^N_3_ (10 atom%, Sigma-Aldrich, Germany). Despite high variability, ^13^C uptake rates calculated as ATP (49 ± 44 nmol g^-1^ fw h^-1^, *n* = 12) and as CTP equivalents (57 ± 38 nmol g^-1^ fw h^-1^, *n* = 12) were similar. The same was found when the ^15^N uptake rates were calculated as ATP (61 ± 45 nmol g^-1^ fw h^-1^, *n* = 12) and CTP equivalents (55 ± 34 nmol g^-1^ fw h^-1^, *n* = 12). This result was irrespective of the addition of MoO_4_^2-^ as acid phosphatase inhibitor or the addition of P_i_ for competition (Figure 7). The relationship between ^13^C uptake and ^15^N uptake as nucleotide equivalents reached a correlation of 1.02 ± 0.03 for ATP and of 0.89 ± 0.04 for CTP (Table 1). These results show that neither the uptake of ^13^C nor the uptake of ^15^ N applied to excised poplar roots as double-labeled ATP or CTP was influenced by the acid phosphatase inhibitor molybdate or by P_i_.

### ^13^C, ^15^N, and ^33^P Uptake From the Respective Labeled ATP by Beech Roots

To compare the results achieved with poplar with another temperate forest tree species, uptake of ^33^P, ^13^C, and ^15^N from labeled ATP was investigated with excised roots from *F. sylvatica* seedlings. In parallel experiments, γ^33^P-ATP and ^13^C/^15^N double-labeled ATP (10 atom% ATP-^13^C_10_^15^N_5_) was applied (Supplementary Figure S2). ^33^P uptake rates equivalent to ATP after γ^33^P-ATP application amounted to 101 ± 31 nmol g^-1^ fw h^-1^ (Supplementary Figure S2). ^33^P uptake from γ^33^P-ATP was affected neither by P_i_ nor by the acid phosphatase inhibitor MoO_4_^2-^. The latter coincide with the findings of poplar (Figure 4A). Xylem loading of ^33^P from γ^33^P-ATP was negligible in beech roots (data not shown). ^13^C and ^15^N uptake rates of beech roots equivalent to ATP (22 ± 7 and 30 ± 15 nmol g^-1^ fw h^-1^, respectively) amounted to one fourth of the ^33^P uptake equivalent to ATP. ^13^C and ^15^N uptake of beech roots as equivalent to ATP was also neither affected by P_i_ nor by the acid phosphatase inhibitor MoO_4_^2-^. The relationship between the ^13^C and ^15^N uptake equivalent to ATP showed a strong correlation of 1.004 ± 0.182 (Supplementary Figure S2) as also observed for poplar roots (Figure 5, 6).

### ^13^C and ^15^N Uptake Rates From ^13^C/^15^N-Labeled ATP of Beech in the Field

**FIGURE 8:**
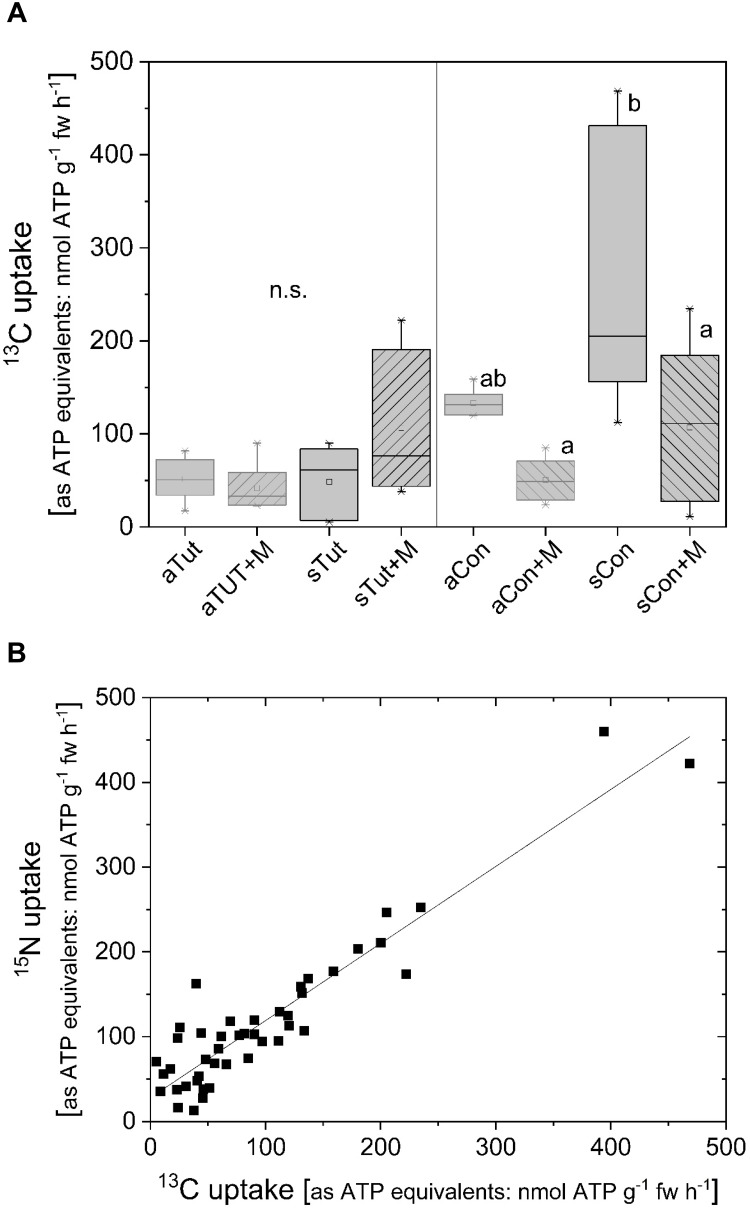
^13^C uptake rates as ATP equivalents of beech roots still attached to adult trees and their offspring at two forest stands low in soil P. Roots of adult beech trees and their offspring at two forests, Tuttlingen (Tut, 9/21/2017) and Conventwald (Con, 9/19/2017) (Netzer et al., 2017), were excavated out of the soil and washed with distilled water. Roots still attached to the adults (*n* = 6) and offspring (*n* = 6) were incubated in an artificial soil solution (adapted to soil water composition of the respective forest site) at pH 5.0 with 0.169 mM ^13^C/^15^N labeled ATP (ATP^13^C_10_/^15^N_5_; 10 atom%). These conditions were selected for comparison reason with the experiments done under controlled conditions. Acid phosphatases were inhibited by the addition of 0.5 mM MoO_4_^2-^. **(A)** ^13^C uptake rates as ATP equivalents of roots from adult beech tress from the Tut (aTut) and from the Con (aCon) forest as well as from the natural regeneration at the Tut (sTut) and Con (sCon) forest. Supplementation of molybdate (0.5 mM, MoO_4_^2-^) is indicated by +M. ^15^N uptake rates as ATP equivalents were comparable to the values received for ^13^C uptake. Statistically significant differences were analyzed by One Way ANOVA followed by the *Post hoc* tests Bonferroni and Tukey with *p* < 0.05. Different small letters for the Con forest indicate statistically differences at *p* < 0.05. At the Tut forest, statistically significant differences were not observed (n.s.). **(B)** The correlation between ^13^C and ^15^N uptake rates as ATP equivalents. Regression characteristics are given in Table 1.

^13^C and ^15^N uptake from double-labeled ATP was furthermore investigated at two beech forest stands characterized as low-P forests (Netzer et al., 2017). Different to the experiments under controlled condition, in the field roots of adult beech trees and their offspring were mycorrhizal and only ^13^C/^15^N double-labeled ATP could be applied to the roots still attached to trees. ^13^C uptake rates as ATP equivalents by beech roots of the extremely low-P forest stand Tut were comparable for adult beech trees and their offspring. Furthermore, inhibition of acid phosphatases by MoO_4_^2-^ did not affect ^13^C uptake rates equivalent to ATP (Figure 8A). In contrast, at the Con forest ^13^C uptake rates were higher compared to the Tut site for both, adult beech trees and their offspring (Figure 8A). At the Con forest, addition of MoO_4_^2-^ to inhibit acid phosphatases caused a decline in ^13^C uptake equivalent to ATP to the level observed for adult trees and their offspring at the Tut forest. ^15^N uptake as ATP equivalent was similar as calculated from the ^13^C uptake equivalent to ATP. Consequently, a strong correlation was found between the ^13^C and ^15^N uptake (0.91 ± 0.05, Table 1 and Figure 8B).

## Discussion

The present study indicates that poplar and beech roots take up P from nucleotides most probably after cleavage of P_i_ although uptake of ADP and/or AMP cannot be excluded. However, the present study also indicates that AMP or at least adenosine can be taken up by tree roots as a whole molecule and contributes not only to P but also to N acquisition of the trees. The common assumption for P acquisition by plants, however, is that plants can take up only P_i_ (Chiou and Lin, 2011). Important P_org_ compounds in the rhizosphere are phosphoric acid anhydrides such as ADP and ATP (Huang et al., 2017), which are hardly detectable in natural environments because of their thermodynamic instability (De Nobili et al., 1996). Nevertheless, it can be assumed that ATP is available around plant roots from dead and destroyed microbial biomass (Lareen et al., 2016) and root exudation (Tanaka et al., 2010, 2014). The latter one led to the abundance of extracellular ATP in regions of active growth and cell expansion at the root surface of *Medicago truncatula* (Kim et al., 2006). Around roots of different plant species, the depletion of P_org_ correlated with acid and alkaline phosphatase activity (Tarafdar and Jungk, 1987). Consequently, P_i_ becomes available for uptake after cleavage from organic bound P (P_org_) by phosphatases (Smith et al., 2015; Tian and Liao, 2015; Hofmann et al., 2016). Recent studies also showed that ecto-apyrases are essential for both, rhizobial and mycorrhizal symbiosis, presumably by modulating extracellular ATP levels (Tanaka et al., 2014). Ecto-apyrases cleave P_i_ from ATP and ADP, but not from AMP (Thomas et al., 1999; Okuhata et al., 2011). Apparently, cleavage of P_i_ from ATP by secreted phosphatases (Liang et al., 2010; Plaxton and Tran, 2011; Tian and Liao, 2015; Liu et al., 2016), ecto-apyrases (Thomas et al., 1999) and extracellular nucleotidases contribute to the extracellular breakdown of ATP into ADP, AMP and/or adenosine. Together with bidirectional transport of ATP and/or one of its degradation product(s) *via* the plasma membrane of root cells, ATP homeostasis can be controlled in the rhizosphere. Simultaneously, these processes contribute to the acquisition of P and N for plant nutrition.

### ‘Pro and Contra’ of ATP, ADP, AMP and/or Adenosine Uptake

^33^P in γ^33^P-ATP can enter the root as intact ATP molecule or as P_i_ after cleavage by phosphatases and/or ecto-apyrases that are commonly present in the rhizosphere (see section “Discussion” above). The acid phosphatase inhibitor MoO_4_^2-^ did not affect ^33^P uptake calculated as ATP equivalents when γ^33^P-ATP was applied to excised non-mycorrhizal poplar or beech roots. This result indicates uptake of the intact γ^33^P-ATP molecule. If this assumption is correct, the labeling position of the ^33^P should not affect ^33^P uptake. However, when the αP of ATP was labeled, ^33^P uptake calculated as ATP equivalents was lower compared to the ^33^P uptake from γ^33^P-ATP. Both, phosphatases and ecto-apyrases can cleave the γP_i_ and βP_i_ unit from ATP, thereby contributing to the control of extracellular ATP abundance (Plesner, 1995; Song et al., 2006; Wu et al., 2007; Clark et al., 2010; Haferkamp et al., 2011). As MoO_4_^2-^ does not inhibit plasma membrane apyrases of plant roots (i.e., Gallagher and Leonard, 1982; Thomas et al., 1999), they can still cleave the γP_i_ and βP_i_ unit from ATP in the presence of molybdate. Hence, it can be concluded that ^33^P_i_ might be cleaved from γ^33^P-ATP by this group of enzymes prior its uptake by poplar roots. This conclusion is also evident from the high offset when the ^33^P uptake from γ^33^P-ATP is compared to the ^13^C uptake expressed as ATP equivalents (Table 1) and is further supported by competition experiments. Addition of P_i_ to γ^33^P-ATP diminished ^33^P uptake by poplar roots, however, not by beech roots. Either P_i_ diluted the ^33^P_i_ pool cleaved from γ^33^P-ATP by phosphatases, ecto-apyrases and/or nucleotidases excreted by the roots or P_i_ functions as competitive inhibitor of ATP uptake by poplar roots; *vice versa*, ATP did not affect P_i_ uptake (Figure 3). For excised beech roots, addition of P_i_ did not affect ^33^P uptake from γ^33^P-ATP, strongly supporting the idea of intact γ^33^P-ATP uptake (Supplementary Figure S2A). However, ^33^P-P_i_ uptake by excised beech roots was diminished in the presence of ATP. Together these findings support the common assumption that P_i_ needs to be cleaved from organic bound P prior P_i_ is taken up by phosphate transporters (Kavka and Polle, 2016, 2017; Versaw and Garcia, 2017), but they also support the idea of nucleotide uptake by nucleotide exchange transporters with different substrate specificity (Haferkamp et al., 2011).

The missing link for establishing nucleotide/nucleoside uptake by tree roots remains the identification of nucleotide transporters located at the root plasma membrane. Although adenine nucleotide transporters are characterized as ATP/ADP exchange carrier proteins at different cellular membranes (Leroch et al., 2008; Linka and Weber, 2010; Haferkamp et al., 2011), information about plasma membrane exchange carriers is scarce. To the best knowledge of the authors, only one report of a plasma membrane located ATP exporter has been published. This transporter is essential during pollen maturation in *Arabidopsis* (Rieder and Neuhaus, 2011) and coincidences with a signaling function of extracellular ATP (Roux and Steinebrunner, 2007; Tanaka et al., 2010, 2014). In contrast to ATP/ADP exchange carrier proteins, which so far have not been reported for the plasma membrane of root cells, nucleoside and nucleobase transporters have been described in a number of studies (Möhlmann et al., 2010; Cornelius et al., 2012; Girke et al., 2014; Niopek-Witz et al., 2014). Hence, after cleavage of all three P_i_ units from ATP by enzymes commonly occurring in the rhizosphere such as phosphatases, ecto-apyrases and/or nucleotidases, the remaining nucleoside adenosine can be taken up as complete molecule.

In the present experiments, the ribose and the base of adenosine were labeled with ^13^C, but only the base carried the ^15^N label (Figure 1). In both, excised beech and poplar roots, ^13^C and ^15^N uptake rates determined as ATP or CTP equivalents, were similar and showed a strong correlation to each other (Table 1). Hence, separate uptake of the nucleobase and the ribose unit after hydrolysis by extracellular nucleoside hydrolases (Jung et al., 2011; Tanaka et al., 2014) seems highly improbable. However, the strong correlation between ^13^C and ^15^N uptake does not indicate whether ADP, AMP and/or adenosine is taken up after cleavage of the γP, βP, and αP. Rather, the offset of the ^15^N uptake observed in all experiments (Table 1) indicates a slightly higher ^15^N uptake compared to ^13^C that can be attributed to the cleavage into ribose and the nucleobase by nucleoside hydrolases (Jung et al., 2011). Whether the base and the ribose units from nucleosides are taken up separately (Riewe et al., 2008; Jung et al., 2011; Tanaka et al., 2014) by nucleobase (Girke et al., 2014) and sugar transporters (Williams et al., 2000) needs further studies.

Still, ^13^C and ^15^N uptake rates by excised poplar roots determined as ATP equivalents decreased, however, not statistically significant, at higher temperatures when MoO_4_^2-^ inhibited extracellular acid phosphatase activity indicating uptake of AMP and/or adenosine after P_i_ cleavage. In addition, if attached roots of adult beech trees and their natural regeneration in the Con forest were exposed to ^13^C/^15^N labeled ATP plus MoO_4_^2-^, ^13^C and ^15^N uptake as ATP equivalents declined. These results indicate uptake of ADP, AMP and/or adenosine after cleavage of at least one P_i_ unit. It is assumed that in the experiments with excised poplar roots higher temperature increased extracellular phosphatase activity and, hence, the cleavage of γP, βP, and αP from ATP. As a result, increasing amounts of ADP, AMP and/or adenosine are available for its uptake by roots. If MoO_4_^2-^ inhibited extracellular acid phosphatase activity under these conditions, P_i_ was not cleaved from ATP and the availability of ADP, AMP and adenosine for root uptake declined; although based on the literature ecto-apyrases upon MoO_4_^2-^ application were not inhibited (Tanaka et al., 2011) and can still cleave P_i_ from ATP and ADP. Thus, the relevance of P_i_ cleavage from ATP by phosphatases and/or ecto-apyrases for P acquisition under field conditions will depend on soil temperature and consequently also on the season, but also on the enzyme composition of the rhizosphere.

Under field conditions, the uptake of ^13^C and ^15^N from the ATP applied furthermore depends on other factors at the forest stand. Tree roots interact with physical, chemical and biological properties of the soil in the rhizosphere (Richardson et al., 2009). Differences of ^13^C and ^15^N uptake rates from ATP between the Tut and the Con forest stands may thus be linked to different soil characteristics of the two forest stands. (i) The soils differ in pH, ranging from 5.7 to 7.5 for the calcareous Tut site and from 3.6 to 4.3 for the silicate Con forest, as well as in plant available soil P_i_ (Tut: 0.03 ± 0.01 μmol L^-1^ and Con: 0.23 ± 0.18 μmol L^-1^) (for detailed soil description see Prietzel et al., 2016; Netzer et al., 2017). Acid phosphatases are highly active at acidic soil conditions (i.e., Bozzo et al., 2002) that are given at the Con forest (Prietzel et al., 2016) and may be of higher importance at the Con compared to the Tut stand. (ii) In addition, the microbial activity and mycorrhizal communities differ between the two study sites (Leberecht et al., 2016a,b; Zavišić et al., 2016), most likely with the consequence of differences in phosphatase secretion (Hofmann et al., 2016). The microbial biomass in the rhizosphere consists of active as well as of inactive and dead microbes and usually is quantified in “static” approaches, mainly based on the single-stage determination of cell components such as ATP, DNA, and RNA (Blagodatskaya and Kuzyakov, 2013). Hence, substantial amounts of ATP should be present in the rhizosphere as a P and N source, which will depend on seasonal and environmental differences affecting microbial activity. (iii) Finally, differences in phosphatase, ecto-apyrase and nucleotidase profiles of the beech rhizosphere between the two forest stands can affect P_i_ cleavage from ATP depending on environmental conditions such as soil P_i_, pH, microbial activity and the season. The lower plant available nitrogen and phosphorus in the soil of the Tut compared to the Con forest (Rennenberg and Dannenmann, 2015) coincided with the lower ^13^C and ^15^N uptake from ATP of beech offspring in the present study. Therefore, it is concluded that the processes described above are highly significant in determining the nutrient availability in forest soils.

## Author Contributions

CH and HR designed the research project. CH wrote the manuscript and supervised all experiments. US performed most of the experiments. NT performed experiments on the temperature influence on ^13^C/^15^N-ATP uptake. FN performed ATP uptake experiments in the field.

## Conflict of Interest Statement

The authors declare that the research was conducted in the absence of any commercial or financial relationships that could be construed as a potential conflict of interest.

## Footnotes

1 www.originlab.com
