# Supplementary material for: ATP as Phosphorus and Nitrogen Source for Nutrient Uptake by Fagus sylvatica and Populus x canescens Roots
Source: Front Plant Sci. 2019 Apr 4;10:378. doi: 10.3389/fpls.2019.00378 (PMC6458296; doi:10.3389/fpls.2019.00378)

ATP as phosphorus and nitrogen source for nutrient uptake by *Fagus sylvatica* and *Populus* x *canescens* roots

**Running title: Nucleotides as sources for P and N acquisition**

Ursula Scheerer^1^, Niclas Trube^1^, Florian Netzer^1,2^, Heinz Rennenberg^1^ and Cornelia Herschbach^1,2^*

^1^Chair of Tree Physiology, Institute of Forest Sciences, Albert-Ludwigs-University Freiburg, Georges-Köhler-Allee 53/54, 79110 Freiburg, Germany

^2^Chair of Ecosystem Physiology, Institute of Forest Sciences, Albert-Ludwigs-University Freiburg, Georges-Köhler-Allee 53/54, 79110 Freiburg, Germany

Corresponding Author:

Prof. Dr. Cornelia Herschbach, Chair of Ecosystem Physiology, Institute of Forest Sciences, Albert-Ludwigs-University Freiburg, Georges-Köhler-Allee 53/54, 79110 Freiburg, Germany, email: [cornelia.herschbach@ctp.uni-freiburg.de](mailto:cornelia.herschbach@ctp.uni-freiburg.de), phone: +49 761 2038303, Fax: +49 761 2038302

**Supporting Information**

Additional Supporting Information can be found online in the Supporting Information tab for this article:

**Supplemental Figure S1.** pH-dependency of phosphate uptake, xylem loading of phosphate and the proportion of P_i_ loaded into the xylem of excised **poplar** roots.

**Supplemental Figure S2.** The effects of acid phosphatase inhibition and of P_i_ competition on ^33^P uptake from γ^33^P-ATP and on ^13^C/^15^N uptake rates as ATP equivalents from ^13^C/^15^N labelled ATP by excised **beech** roots.

**Supplemental Figure S1.** pH-dependency of phosphate uptake, xylem loading of phosphate and the proportion of P_i_ loaded into the xylem of excised **poplar** roots applied as ^33^P-P_i_.

The pH dependency of phosphate uptake (upper graph), xylem loading of phosphate (middle graph) and the relative portion of phosphate loaded into the xylem (bottom graph) was carried out with excised roots from poplar plants grown with 0.25 mM P_i_ (^33^P-P_i_ during incubation at different pH values was 0.25 mM P_i_ with ~2.8*10^9^ Bq mmol^-1^). Data presented are mean values ± SD (n = 4 to 8) from individual incubations with 4 to six excised poplar roots. Statistically significant differences for the P_i_ uptake rates, the P_i_ loaded into the xylem and for the proportion of P_i_ loaded into the xylem were not found. Xylem loading of P_i_ was comparable across all pH values and ranged from 0.5 to 4.5 nmol g^-1^ fw h^-1^. The proportion of P_i_ loaded into the xylem reached a maximum of 4%.


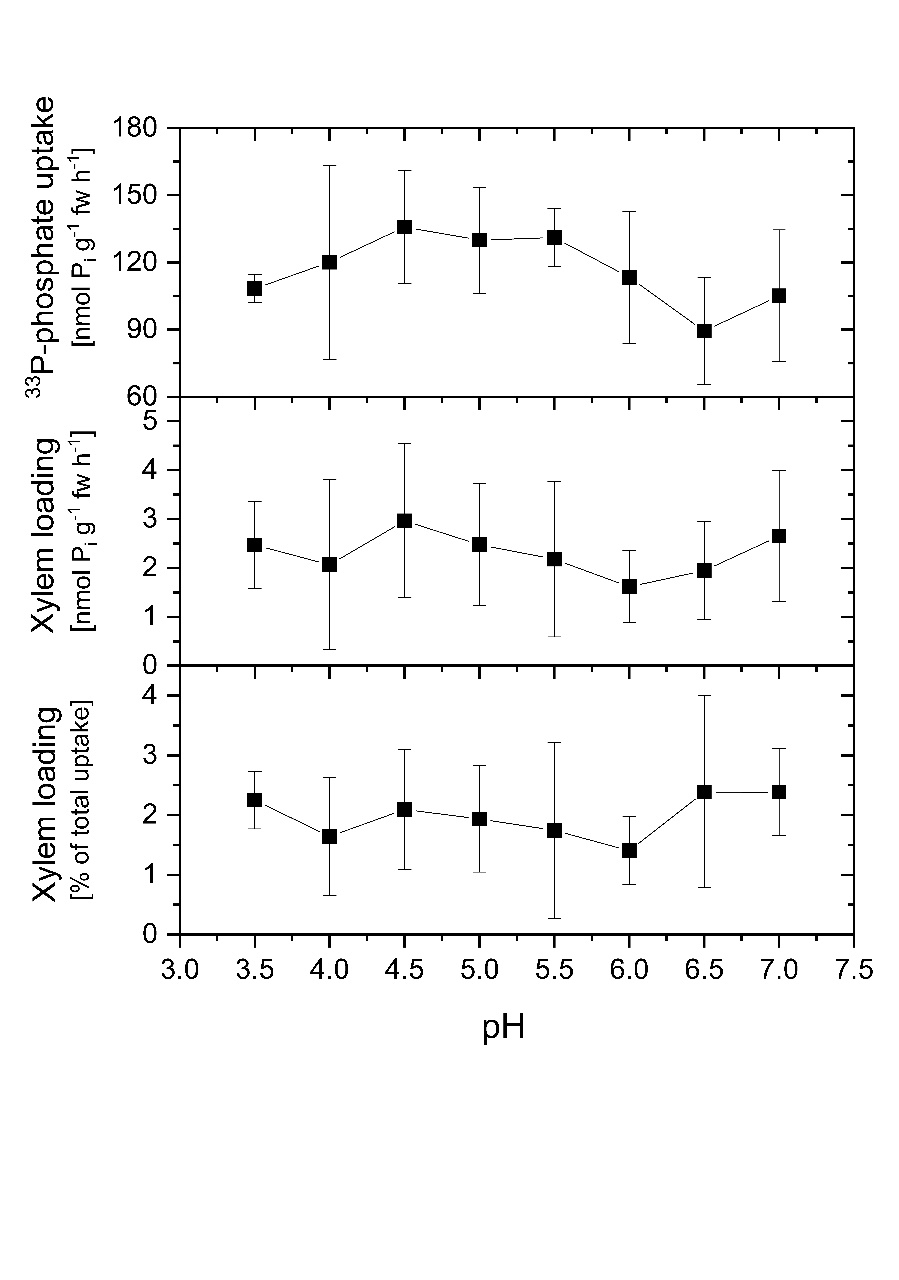


**Supplemental Figure S2.** The effects of acid phosphatase inhibition and of P_i_ competition on ^33^P uptake from γ^33^P-ATP and on ^13^C/^15^N uptake rates as ATP equivalents from ^13^C/^15^N labelled ATP by excised **beech** roots.

Excised roots were taken from two years-old beech seedling cultivated by a gardener, adapted greenhouse conditions and treated with tap water. Excised beech roots were taken in July (n = 4) and September (n = 4), exposed for 4 h to 0.169 mM ATP either applied as γ^33^P-ATP (~5.6*10^7^ Bq mmol^-1^ ATP) (**A**) or as ^13^C/^15^N labelled ATP (ATP-^13^C_10_/^15^N_5_, 10 atom%) (**B**, **C**). The P_i_ concentration during incubation was 0.25 mM and the MoO_4_^2-^ concentration was 0.5 mM. ^33^P uptake rates as ATP equivalents were calculated based on the ^33^P incorporation (**A**). ^13^C (**B**) and ^15^N uptake rates (**B**, **C**) were calculated in ATP equivalents from ^15^N and ^13^C incorporation. (**C**) The relationship between ^13^C and ^15^N uptake rates as ATP equivalents over all treatments. The slope is 1.00 ± 0.18, the y intercept is 3.1 ± 5.3, Pearson is 0.847 and the correlation coefficient is 0.717.


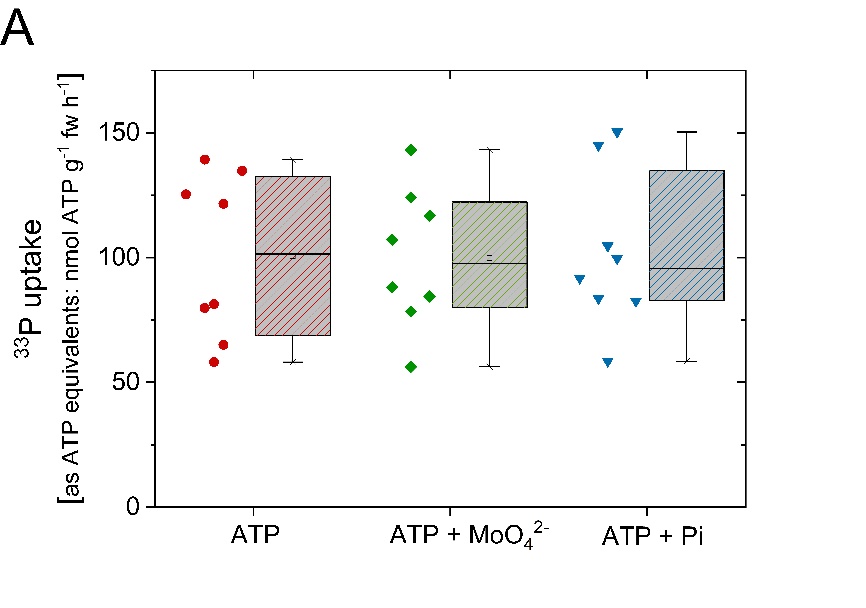

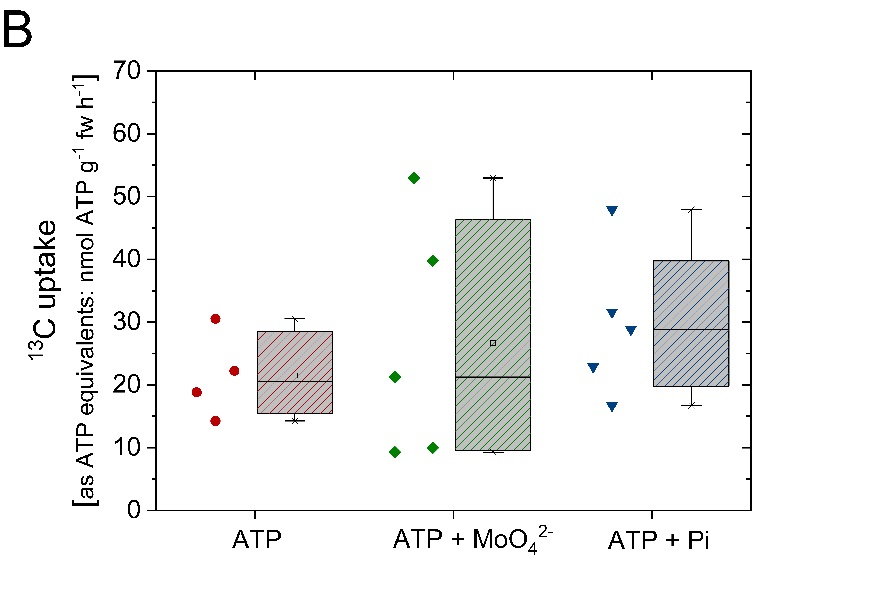

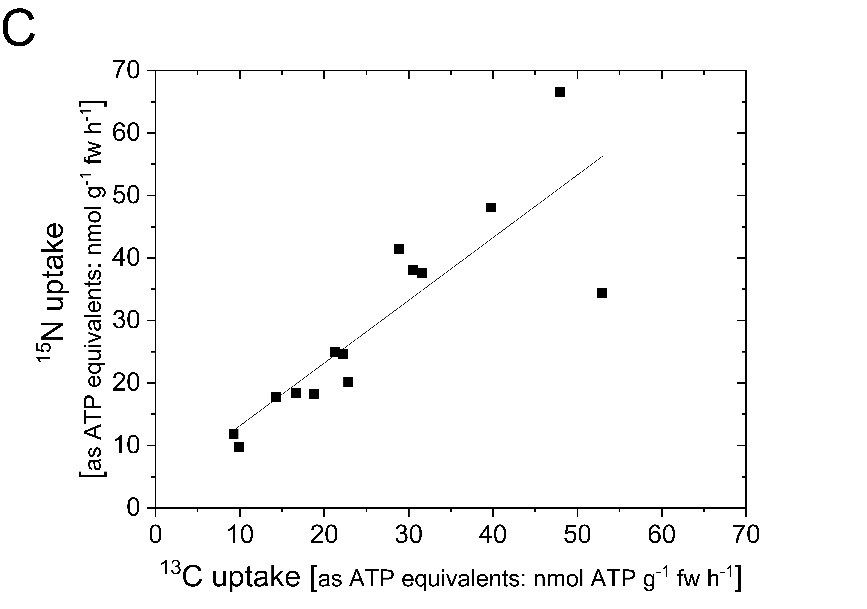

Supplement: Supplementary file 1 [file Data_Sheet_1.docx]
